# Chronic Suppurative Osteomyelitis of Subcondylar Region: A Case Report

**DOI:** 10.5005/jp-journals-10005-1202

**Published:** 2013-08-26

**Authors:** G Ravi Kumar, Basheer Ahmed Syed, N Prasad, SP Praveen

**Affiliations:** Assistant Professor, Department of Pedodontics and Preventive Dentistry, Government Dental College and Hospital, Hyderabad Andhra Pradesh, India, e-mail: drravimds@gmail.com; Associate Professor, Department of Prosthodontics and Crown and Bridge Government Dental College and Hospital, Rajiv Gandhi Institute of Medical Sciences, Kadapa, Andhra Pradesh, India; Assistant Professor, Department of Oral and Maxillofacial Surgery Government Dental College and Hospital, Hyderabad, Andhra Pradesh, India; Associate Professor, Department of Oral and Maxillofacial Surgery Government Dental College and Hospital, Rajiv Gandhi Institute of Medical Sciences, Kadapa, Andhra Pradesh, India

**Keywords:** Osteomyelitis, Chronic suppurative, Sequestra, Surgical debridement

## Abstract

Chronic suppurative osteomyelitis (CSO) of the maxillofacial region is primarily caused by infections of odontogenic microorganisms. It may also arise as a complication of dental extractions, maxillofacial trauma, inadequate treatment of a fracture and irradiation to the mandible. This condition is characterized by areas of devitalized bone (sequestra) which serves as a nidus for recurrent episodes of infection. This case report describes a case of CSO in an untreated right subcondylar fracture of the mandible which was successfully treated with a combination of antibiotic therapy and surgical debridement in an 8-year-old boy.

**How to cite this article:** Kumar GR, Syed BA, Prasad N, Praveen SP. Chronic Suppurative Osteomyelitis of Subcondylar Region: A Case Report. Int J Clin Pediatr Dent 2013;6(2): 119-123.

## INTRODUCTION

Osteomyelitis is an inflammation of bone and bone marrow that develops in the jaws usually after a chronic infection.^1^It may be classified as acute, subacute or chronic osteomyelitis depending on the clinical presentation. Osteomyelitis of the mandible is rare in developed countries and can be attributed to increased availability of antibiotics and higher standards of oral health. The incidence of osteomyelitis may increase in remote rural and regional centers in developing countries because of inadequate access to oral health care professionals. The typical age of presentation is in the fifties to the sixties with males more likely to be affected. Predisposing factors include radiotherapy (osteoradionecrosis), immunocompromised, uncontrolled diabetes and patients on immunosuppressive therapy.^2,3^

In children, chronic osteomyelitis may also be seen after traumatic injuries or as a complication of surgical procedures. Chronic suppurative osteomyelitis (CSO) can only be treated successfully by a combination of antimicrobial therapy with surgery–either sequestrectomy or decortication of the affected bone.^4^ The aim of the surgery is to eliminate all the infected and necrotic bony tissue and if incomplete may lead to persistence of the osteomyelitis. Our goal is to review the treatment of chronic osteomyelitis in an 8-year-old boy.

## CASE REPORT

An 8-year-old boy was referred to the Department of Pediatric Dentistry, Government Dental College and Hospital, Rajiv Gandhi Institute of Medical Sciences, Kadapa, Andhra Pradesh, India, with a 2 years history of discharging pus on right side of the lower jaw.

The past history from the parents revealed that patient had a trauma to the lower jaw 2 years back and was not taken immediate care. After a couple of months after the injury, he developed recurrent painful extraoral swelling over the right side of the angle of the mandible which was temporarily subsided on medication prescribed by a rural medical practioner. Later, it resulted in cutaneous sinus tracts openings and was referred to our department for further evaluation. The patient and their family members belong to a low socioeconomic status with inadequate access to oral health care professionals.

## CLINICAL FEATURES

**Fig. 1:**
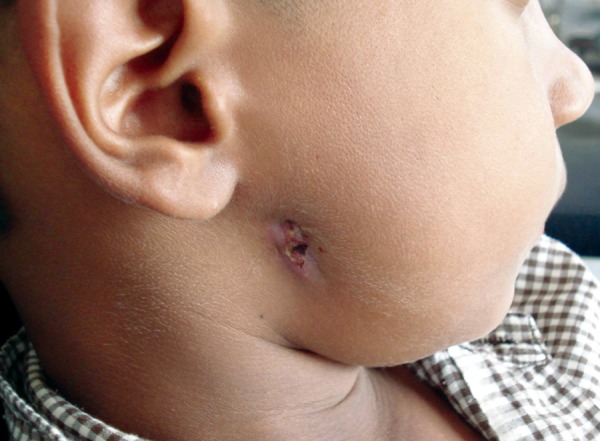
Lateral view of the right side of the face showing the cutaneous sinus openings

**Figs 2A and B:**
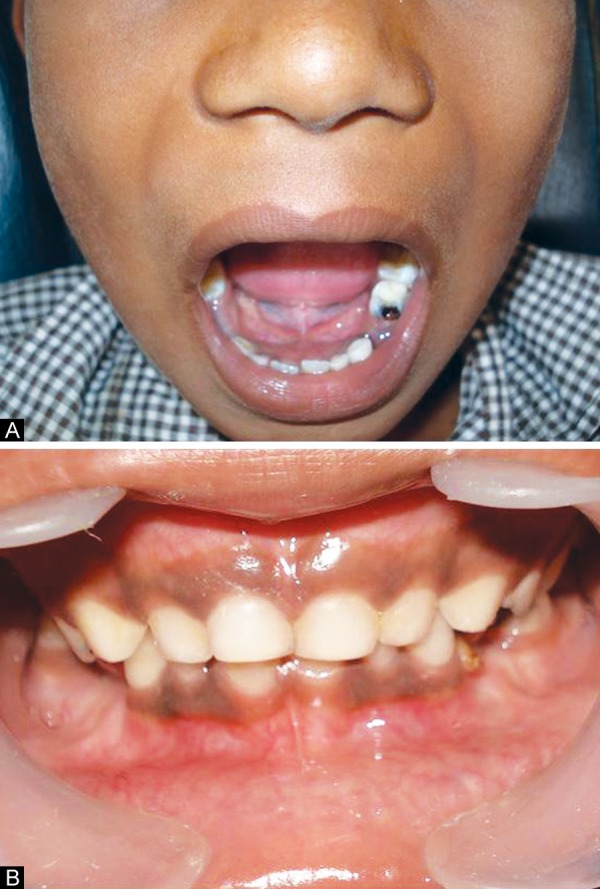
Preoperative intraoral view showing normal occlusion with no midline deviation

On extraoral examination the patient was asymptomatic, afebrile and there was no regional lymphadenopathy. The cutaneous sinus openings (Fig. 1) were two in number and were oval in shape, measuring 2 × 2 mm and 4 × 4 mm placed one over the above were present on the right side of the angle of mandible. The surrounding skin adjacent to the sinus was erythematous and was slightly tender on palpation. Mouth opening appeared to be normal with no midline deviation (Fig. 2A and B). His medical history was noncontributory.

On intraoral examination mixed dentition period with normal occlusion with several decayed primary teeth was noted. His oral hygiene was average and his dental history was found to be insignificant. These clinical features were typical of chronic suppurative osteomyelitis and a clinical diagnosis of CSO of the mandible was made.

### Investigations

**Figs 3A and B:**
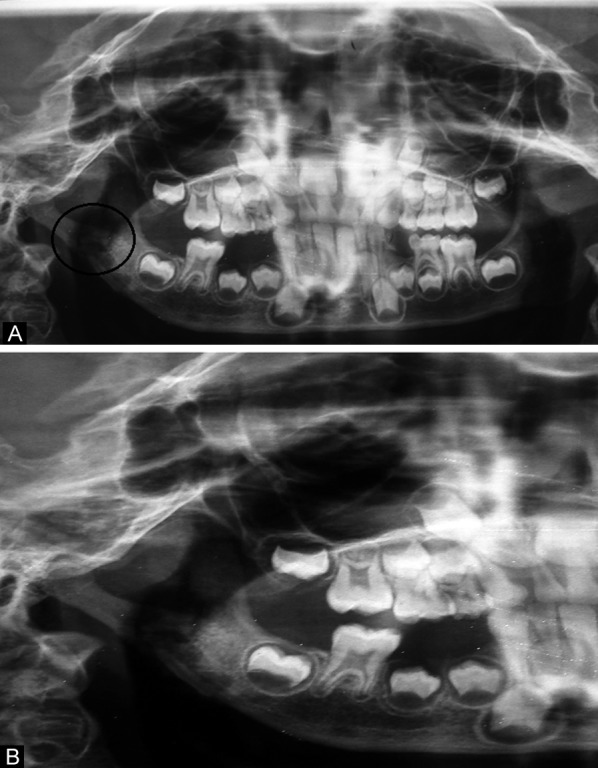
OPG demonstrating osteolysis and sequestra in relation to the right subcondylar region

On microbiological examination *Staphylococcus aureus* was isolated from the pus culture collected in the cutaneous sinus tract. Leukocytosis and elevation of erythrocyte sedimentation rate was noted. Orthopantomogram and lateral oblique view of the mandible revealed osteolysis and sequestra (segments of necrotic bone separated from living bone by granulation tissue) in relation to the right subcondylar region (Fig. 3A and B). Microbiological and radiological findings confirmed the CSO involving right subcondylar region.

### Treatment Procedure

**Fig. 4:**
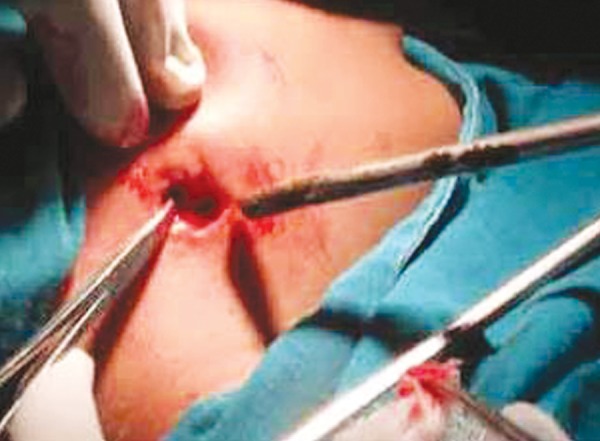
Surgical debridement

**Fig. 5:**
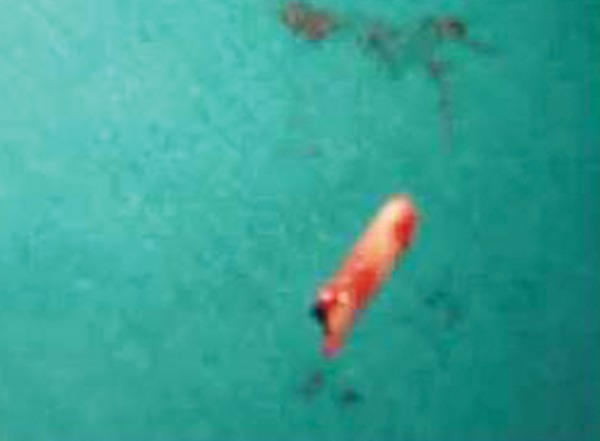
Sequestrum

**Fig. 6:**
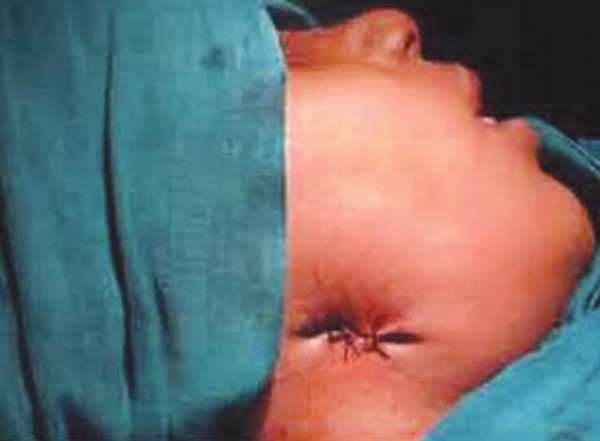
Closure of surgical debrided area

**Fig. 7:**
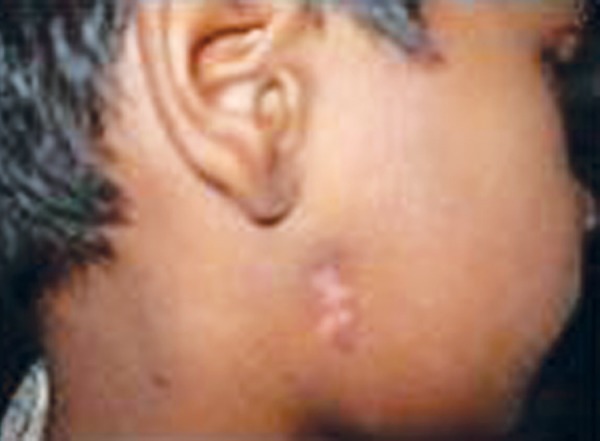
Postoperative lateral view of the face showing the healed cutaneous sinus openings

**Fig. 8:**
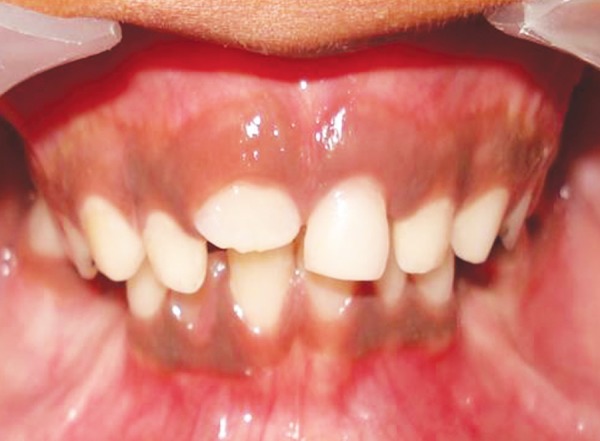
Intraoral view showing the occlusion after the 3 months of surgical debridement

**Fig. 9A and B:**
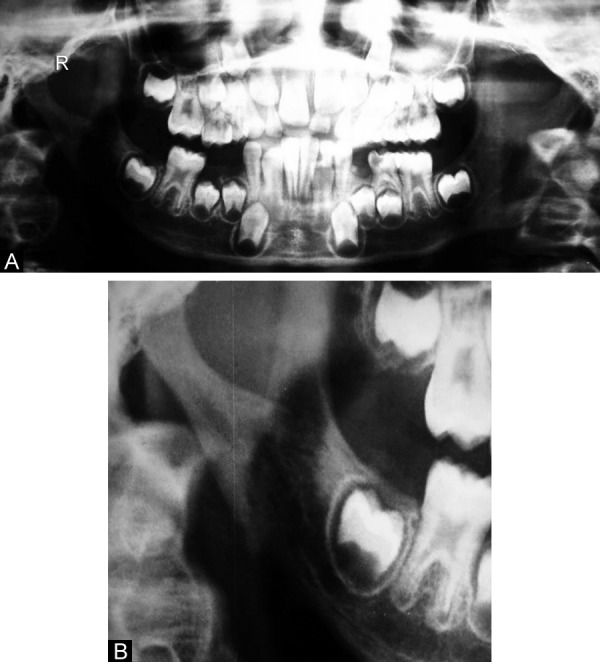
Three months after the postoperative OPG revealing there was no radiological evidence of residual infection

Treatment included a presurgical course of antibiotics (clindamycin 150 mg, Orally qid for 2 weeks) followed by surgical debridement and resection of the cutaneous sinus tract (Fig. 4 to 6) under general anesthesia and the patient was kept on soft and liquid diet for 3 to 4 weeks to prevent the risk of fracture of weakened subcondylar bone. Specimens were taken for bacterial cultures and antibiotic sensitivity testing and the resected tissue was sent for histopathological review. Clindamycin was chosen because of its broad antibacterial activity against anaerobic organisms commonly present in chronic ‘mixed’ odontogenic infections and it has the potential to penetrate well and achieve high therapeutic concentrations in bone. Three months later radiographs were repeated. There was no clinical or radiological evidence of residual infection (Fig. 7 to 9).

## DISCUSSION

This case report demonstrates the typical features of CSO and its management a rare but well-described potential complication particularly in children that developed secondary to traumatic injury of the mandible. CSO can be treated successfully by a combination of antimicrobial therapy with surgery–either sequestrectomy or decortication of the affected bone.^3^

Chronic suppurative osteomyelitis is best managed with careful evaluation, staging and establishment of microbial etiology, susceptibilities and treatment includes antimicrobial therapy and debridement with management of resultant dead space and if necessary stabilization of Bone.^5^ The specific microorganism(s) isolated from patients with bacterial osteomyelitis is often associated with the age of the patient or the clinical scenario. *Staphylococcus aureus* is implicated in 90% of the cases of osteomyelitis.^6^*Staphylococcus epidermidis* S. aureus, Pseudomonas aeruginosa, Serratia marcescens *and* Escherichia coli *are also isolated in patients with chronic osteomyelitis.* Staphylococcus aureus is implicated in most cases of acute hematogenous osteomyelitis and is responsible for up to 90% of cases; this infection occurs predominantly in children and is often seeded hematogenously. The most common site is the rapidly growing and highly vascular metaphysis of growing bones. In adults, osteomyelitis is usually a subacute or chronic infection that develops secondary to an open injury to bone and surrounding soft tissue.

In most patients with osteomyelitis early antibiotic therapy produces the best results. The minimum duration of antibiotic therapy to treat CSO is 2 weeks.^7^ However, it has been suggested by Bamberger that a minimum of 4 weeks is indicated to achieve an acceptable rate of cure.^8^In the present case, the patient was prescribed a 2-week course of oral clindamycin in combination with surgical debridement. However, without adequate debridement chronic osteomyelitis does not respond to most antibiotic regimens. The aim of surgery is to eradicate all infected/ devascularized tissue because antibiotics cannot penetrate devascularized tissue.^9^ The quality of the debridement is the most critical factor and if incomplete may lead to persistence of the osteomyelitis. It is also essential to improve the host's physiological state through adequate nutrition, correction of significant anemia if present and treating any coexisting infectious disease. The timing of surgical intervention is controversial. Some authors recommend early sequestrectomy to provide a better environment for the periosteum to respond.^10^ Others recommend to wait until a sufficient involucrum has formed before performing a sequestrectomy to minimize the risk of complications such as fracture, nonunion, deformity, and segmental bone loss. In either case it is critical to preserve the involucrum.

Ideally, the debridement can be carried out through a window in the weakest area of the involucrum to maximize structural integrity. Sinus tracts and any adjacent scar tissue are resected during the initial skin exposure. Injection of contrast material (sinogram) or methylene blue preoperatively may help to better define the anatomy of a sinus.^11^ Although the extraperiosteal exposure may be wide, the subperiosteal exposure should be limited to the area of bone to be removed to preserve the local blood supply. All sequestra should be removed and any devitalized tissue should be curetted. The extent of debridement may be defined by the presence of punctate bleeding from the exposed bony surface referred to as the ‘paprika sign’.^12^Once all abnormal tissue has been removed the wound should be thoroughly irrigated with sterile saline with or without an antibiotic solution. Boiled or distilled water is also an effective irrigant. The wound should be closed loosely to allow for drainage. There should be no tension on the skin edges. The bone edges are sculpted to facilitate soft tissue coverage. If possible muscle may be mobilized to cover the exposed bony surfaces.^13^ A drain should be placed after debridement with excision of bone to obliterate the dead space created by the removal of tissue. Dead space management includes local myoplasty, free-tissue transfers and the use of antibiotic impregnated beads. Soft tissue procedures have been developed to improve local blood flow and antibiotic delivery.

### Follow-up

Early antibiotic therapy before extensive destruction of bone produces the best results in patients with osteomyelitis. During treatment patients should be followed closely for signs and symptoms of worsening infection. After the completion of treatment follow-up should be based on the response to therapy and the overall health of the patient.

## References

[1] Bernier S, Clermont S, Maranda G, Turcotte JY (1995). Osteomyelitis of the jaws.. J Can Dent Assoc.

[2] Hudson JW (1993). Osteomyelitis of the jaws: A 50-year perspective.. J Oral Maxillofac Surg.

[3] Aitasalo K, Niinikoski J, Grénman R, Virolainen E (1998). A modified protocol for early treatment of osteomyelitis and osteoradio- necrosis of the mandible.. Head Neck.

[4] van Merkesteyn JP, Groot RH, van den Akker HP, Bakker DJ, Borgmeijer-Hoelen AM (1997). Treatment of chronic suppurative osteomyelitis of the mandible.. Int J Oral Maxillofac Surg.

[5] Cierny G, Mader JT (1983). The surgical treatment of adult osteomyelitis. In: Evarts, CM., editors. Surgery of the musculoskeletal system. Vol. 10..

[6] Cole WG, Dalziel RE, Leitl S (1982). Treatment of acute osteomyelitis in childhood.. J Bone Joint Surg Br.

[7] Marx RE (1992). Chronic osteomyelitis of the jaws. In: Laskin, D.; Strass, R., editors. Oral and maxillofacial surgery clinics of North America..

[8] Bamberger DM (1993). Osteomyelitis. A common sense approach to antibiotic and surgical treatment.. Postgrad Med.

[9] Simpson AH, Deakin M, Latham JM (2001). Chronic osteomyelitis. The effect of the extent of surgical resection on infection-free survival.. J Bone Joint Surg Br.

[10] Daoud A, Saighi-Bouaouina A (1989). Treatment of sequestra, pseudarthroses, and defects in the long bones of children who have chronic hematogenous osteomyelitis.. J Bone Joint Surg Am.

[11] Fowles JV, Lehoux J, Zlitni M, Kassab MT, Nolan B (1979). Tibial defect due to acute haematogenous osteomyelitis: Treatment and results in twenty-one children.. J Bone Joint Surg Br.

[12] Tetsworth K, Cierny G 3rd (1999). Osteomyelitis debridement techniques.. Clin Orthop Relat Res.

[13] Bosworth DM, Liebler WA, Natstasi AA (1966). Resection of the tibial shaft for osteomyelitis in children. A thirty-two-year follow- up study.. J Bone Joint Surg Am.

